# Awareness of primary immunodeficiency diseases at a national pediatric reference center in Peru

**DOI:** 10.31744/einstein_journal/2021AO6289

**Published:** 2021-12-03

**Authors:** Liz Eliana Veramendi-Espinoza, Jessica Hanae Zafra-Tanaka, Crhistian Toribio-Dionicio, Mariella R. Huamán, Gabriela Pérez, Wilmer Córdova-Calderón

**Affiliations:** 1 Department of Allergy Primary Immunodeficiency Unit Hospital Nacional Edgardo Rebagliati Martins Lima LIM Peru Department of Allergy, Primary Immunodeficiency Unit, Hospital Nacional Edgardo Rebagliati Martins, Lima, LIM, Peru.; 2 Center of Excellence in Chronic Diseases Universidad Peruana Cayetano Heredia Lima LIM Peru Center of Excellence in Chronic Diseases, Universidad Peruana Cayetano Heredia, Lima, LIM, Peru.; 3 Faculty of Medicine Universidad Nacional Mayor de San Marcos Lima LIM Peru Faculty of Medicine, Universidad Nacional Mayor de San Marcos, Lima, LIM, Peru.; 4 Sociedad Cientifica de San Fernando Universidad Nacional Mayor de San Marcos Lima LIM Peru Sociedad Cientifica de San Fernando, Universidad Nacional Mayor de San Marcos, Lima, LIM, Peru.; 5 Unidad Funcional de Alergia, Asma e Inmunologia Instituto Nacional de Salud del Niño Breña LIM Peru Unidad Funcional de Alergia, Asma e Inmunologia, Instituto Nacional de Salud del Niño, Breña, LIM Peru.

**Keywords:** Awareness, Education, medical, Primary immunodeficiency diseases/diagnosis, Peru

## Abstract

**Objective:**

To investigate the level of awareness of primary immunodeficiency diseases among physicians working at *Instituto Nacional de Salud del Niño*.

**Methods:**

Cross-sectional study including pediatric residents and pediatricians working at the *Instituto Nacional de Salud del Niño* during the study period (2017-2019). Physicians working at the immunology unit and surgery departments were excluded. Three aspects of awareness of primary immunodeficiency diseases were investigated: education, general knowledge, and diagnostic suspicion and actions taken in the face of suspicion.

**Results:**

This sample comprised 83 physicians with a median age of 33 years. Most physicians were women (71.1%) and half were pediatric residents. During their undergraduate studies, 43.1% had taken primary immunodeficiency disease courses, and 39.2% had attended conferences on this topic. During their residency training, 25.9% had taken primary immunodeficiency disease courses, and 60.3% had participated in conferences on this topic. Among pediatricians, 50% had taken primary immunodeficiency disease courses, and 53.1% had attended conferences on this topic. Only 39.8% of physicians reported being familiar with the list of 10 warning signs developed by the Jeffrey Modell Foundation. More than half of physicians considered the lack of access to laboratory tests the major challenge in making diagnosis of primary immunodeficiency diseases.

**Conclusion:**

This study revealed limited awareness of primary immunodeficiency diseases among physicians working at *Instituto Nacional de Salud del Niño*. Although most physicians suspected primary immunodeficiency diseases in patients with a history of recurrent infections and frequent use of antibiotics, not all of them were familiar with the list of 10 warning signs proposed by the Jeffrey Modell Foundation, nor were they able to describe ancillary tests requested in suspected cases.

## INTRODUCTION

Primary immunodeficiency diseases (PIDD) are a heterogeneous group of disorders of the immune system. The most common presentations include severe recurrent infections, neoplasms, atopy and autoimmune conditions, with high mortality, especially in patients with severe combined immunodeficiency.^(1,2)^ Approximately 6 million people worldwide suffer from PIDD.^(3)^ However, the number of reported cases is lower.

In August 2020, the Latin American Society for Immunodeficiencies (LASID) registered 8,383 cases of PIDD. Peru contributed with 213 cases (2.5% of total caseload), of which 33.8% were reported by *Unidad Funcional de Alergia, Asma e Inmunologia* (UFAAI), of the *Instituto Nacional de Salud del Niño* (INSN) - Breña, LIM, Peru^(4)^ the most important Peruvian health center for training in pediatrics. In 2015, the Jeffrey Modell Foundation recognized the UFAAI and two other immunology centers as Jeffrey Modell Foundation Diagnostic and Research Centers, a crucial step in establishing international collaborations for the benefit of Peruvian PIDD patients. The number of registered cases in Latin America has been on the rise year after year due to improved diagnostic techniques and enhanced awareness.^(5,6)^ However, many challenges must still be overcome in order to guarantee coverage for a higher number of PIDD patients.^(7,8)^

Different studies suggest low awareness of PIDD among physicians may delay diagnosis and treatment of PIDD patients. According to a study performed in the United States, awareness of PIDD was limited to 32% of physicians and was greater among pediatricians.^(9)^ A Brazilian study revealed that, although 80% of physicians admitted recurrent infections may be related to PIDD, only 40% evaluated these patients.^(10)^ Likewise, only 32% of physicians approved a survey of PIDD awareness in Iran. Even though the highest scores were attributed to treatment, 86% of physicians reported having problems in managing these patients.^(11)^

Knowledge and awareness have different meanings, the latter being mainly related to attitude and action.^(6,9-11)^ In that sense, lack of awareness among physicians may explain the limited number of reported cases of PIDD, and the delayed diagnosis in Latin American countries, such as Peru.^(4,12)^ This study aimed to investigate the level of awareness of PIDD among physicians working at a national pediatric reference center in Peru. Findings of this study may inform the development of strategies aimed to increase awareness of PIDD, which may contribute to early diagnosis and timely treatment provision for affected patients.

## OBJECTIVE

To investigate the level of awareness of primary immunodeficiency diseases among physicians working at *Instituto Nacional de Salud del Niño*.

## METHODS

### Study design and population

Cross-sectional study conducted at INSN. The study population comprised all physicians working at INSN during the study period (October 2017- March 2019). Pediatric residents and pediatricians working at pediatric inpatient units were included. Physicians working at UFAAI were excluded given their formal training in PIDD. Surgeons were also excluded due to limited access to such specialists.

### Procedures

Initially, random sampling stratified by level of specialization (pediatric resident or pediatrician) was used. The population comprised 148 physicians (105 residents and 43 pediatricians). According to estimations obtained using Epidat V. 4.0 software, a sample size of 106 (75 residents and 31 pediatricians) would be required to yield 40.3% of physicians who had already examined one patient with PIDD,^(10)^ with 5% accuracy and a level of significance of 5%.

A list including all physicians working at INSN at the beginning of this study was used to identify potential participants. Selected physicians were visited twice and invited to participate. However, several potential participants refused to answer the survey due to lack of knowledge about the topic, while others were difficult to contact or locate. For these reasons, convenience sampling was used and physicians were selected during five visits to the hospital complex.

### Study variables

Three aspects related to awareness of PIDD were investigated: education, general knowledge and diagnostic suspicion and actions taken in the face of suspicion.

Educational aspects included questions about PIDD-related courses, conferences, and rotations during undergraduate studies and residency training, or following residency completion.

General knowledge was assessed using the following questions: “Are you aware that patients who often take antibiotics may have primary immunodeficiency diseases?”, “ Do you think primary immunodeficiency diseases are amenable to treatment?”, and “Do you think patients with primary immunodeficiency diseases suffer from a severe disease?”. Response alternatives were “No, never,” “Yes, in some cases,” “Yes, in approximately half of cases,” and “Yes, always.”

In order to assess the ability to suspect PIDD, physicians were asked to list the 10 warning signs of PIDD developed by the Jeffrey Modell Foundation,^(13)^ to evaluate each one of them, and group them accordingly. Difficulties encountered by physicians in obtaining a diagnosis of PIDD were also explored. The 10 warning signs were used due to their originality and scope.^(6)^ To better understand actions taken in the face of PIDD suspicion, physicians were asked whether they had ever examined a patient for PIDD, which laboratory and imaging tests they used, and whether they knew whom to contact for consultation.

The following sociodemographic variables were measured: age, sex, level of specialization (pediatric resident or pediatrician) and years of education.

### Instrument

An *ad hoc* questionnaire including sociodemographic variable- and PIDD-related questions (education, general knowledge and diagnostic suspicion, and actions taken in the face of suspicion) was developed. The questionnaire was validated by four PIDD specialists and one statistician, and tested through interviews with four Peruvian physicians (pediatric residents and pediatricians) who did not participate in the study, and whose suggestions were considered in the development of the final version (Appendix 1).

### Data analysis plan

Categorical variables were described as absolute and relative frequencies. Quantitative variables were described using measures of central tendency and dispersion. Statistical analyses were performed using Stata v14 software (StataCorp LP, 2015, College Station, Texas, USA), with a level of significance of 5%.

### Ethical aspects

This study was reviewed and approved by the Institutional Review Board of INSN number 0287-2018. Participants received an informed consent form explaining the objectives of the study when invited to participate. Information collected was kept confidential.

## RESULTS

This sample comprised 83 physicians with median age of 33 years. Most physicians (71.1%) were females and half of them were pediatric residents. With regard to education at the undergraduate level, 43.1% reported having taken PIDD-specific courses, 39.2% reported having attended PIDD conferences, and only 2% reported having completed a rotation in an immunology unit. With regard to education during the residency training program, 25.9% reported having taken PIDD-specific courses, 60.3% reported having attended PIDD conference, and only 3.5% reported having completed a rotation in an immunology unit.

Pediatricians were questioned about continuing education (*i.e.*, education after completion of residency training in pediatrics). In this group, 50% reported having taken PIDD-specific courses, 53.1% reported having attended PIDD conferences, and only 3.1% reported having completed a rotation in an immunology unit. General knowledge about PIDD among physicians is described in table 1.

**Table 1:** General knowledge about primary immunodeficiency diseases

	Never n (%)	Some cases n (%)	About half n (%)	Always n (%)
Are you aware that patients who often take antibiotics may have primary immunodeficiency diseases?	4 (4.8)	55 (66.3)	20 (24.1)	4 (4.8)
Do you think primary immunodeficiency diseases are amenable to treatment?	5 (6.1)	42 (51.2)	24 (29.3)	11 (13.4)
Do you think patients with primary immunodeficiency diseases suffer from a severe disease?	1 (1.2)	35 (43.2)	28 (34.6)	17 (20.9)

In this sample, 39.8% (n=33) of physicians reported being familiar with the 10 warning signs proposed by Jeffrey Modell Foundation (Table 2).^(13)^ Infection-related signs were the most commonly described, with 33.7% (n=28) of physicians identifying other signs, including recurrent and opportunistic infections (78.6%, n=22), unspecified gastrointestinal disease (35.7%, n=10) and hematologic diseases, such as immune-mediated cytopenia (21.4%, n=6). Furthermore, 94.9% (n=74) of physicians declared live vaccines should be avoided in PIDD patients, and 92.6% (n=75) thought inactivated vaccines were safe.

**Table 2:** List of 10 warning signs (Jeffrey Modell Foundation) reported by physicians

Warning sign	n (%)
≥4 new ear infections within one year	23 (69.7)
≥2 pneumonias within one year	20 (60.6)
Recurrent, deep abscesses in skin or organs (*e.g*.: liver, lungs)	19 (57.6)
≥2 severe sinusitis within one year	17 (51.5)
Failure to gain weight or thrive in infants	17 (51.5)
≥2 deep-seated infections (*e.g*.: sepsis, meningitis)	16 (48.5)
≥2 months on antibiotics with little response	12 (36.4)
Family history of primary immunodeficiency disease	9 (27.3)
Need of intravenous antibiotics to clear infections	5 (15.2)
Persistent oral candidiasis or fungal skin infection	3 (9.1)

Most physicians reported having seen patients with a history of recurrent infections (96.4%, n=80) and frequent use of antibiotics (92.8%, n=77) (Table 3). More than half of physicians (64.6%, n=53) reported having suspected PIDD and requested ancillary tests. However, only 69.8% (n=37) were able to described the tests ordered (Table 4). With regard to actions taken in the face of suspicion of PIDD, 74.7% (n=62) of physicians reported having referred patients to a specialist or specialized organization. In all cases, UFAAI was the referral unit of choice, followed by other hospitals (9.7%, n=6) and other specialists (11.3%, n=7).

**Table 3:** General practice experience reported by physicians

	Never n (%)	≥1/year n (%)	≥1/month n (%)	≥1/week n (%)
Do you see patients with recurrent infections?	3 (3.6)	36 (43.4)	36 (43.4)	8 (9.6)
Do see patients who frequently take antibiotics?	6 (7.2)	32 (38.6)	35 (42.2)	10 (12.1)

**Table 4:** Ancillary tests ordered by physicians (n=37)

Ancillary test	n (%)
Immunoglobulins	36 (97.3)
Lymphocyte count	33 (89.2)
Complete blood count	21 (56.8)
Radiographs, CT, MRI	12 (32.4)
Culture/biopsy	5 (13.5)
HIV testing	4 (10.8)
Neutrophil oxidative burst	4 (10.8)
Complement	3 (8.1)
Exome sequencing	2 (5.4)
Others	19 (51.4)

CT: computed tomography; MRI: magnetic resonance imaging; HIV: human immunodeficiency virus.

When questioned about challenges involved in diagnosis of PIDD, physicians ranked first the lack of access to laboratory tests (57.7%), followed by high test costs (32.1%), and shortage of immunology specialists (5.1%).

## DISCUSSION

In this sample, less than half of participants had taken courses, attended conferences or completed a rotation in an immunology unit during their undergraduate education studies. The number of physicians who attended PIDD-related meetings increased during and after residency. Overall, the number of rotations in immunology units was low. Conversely, a prior study involving 27 general hospitals in Brazil reported pediatricians learned about PIDD throughout their residency training or thereafter (72.2% and 75.9%, respectively).^(10)^ The fact that immunology is a new medical specialty in Peru and that the UFAAI was inaugurated recently, in 2012, may explain this difference, since physicians trained prior to that year may not have had access to appropriate education about PIDD.

This study revealed physicians have limited knowledge about PIDD. Similar findings have been reported in previous studies assessing PIDD-specific knowledge in different countries, such as the United States, Iran, the United Arab Emirates, and Mexico. These studies have shown low levels of information among general practitioners, primary care physicians and pediatricians, including residents.^(9,14-16)^ Interestingly, the fact that these studies have been conducted 5 to 10 years prior to this analysis suggests this is a persistent problem in many countries, regardless of socioeconomic status.

As to warning signs, those associated with infections, which are typical of PIDD, were identified more frequently.^(1)^ However, many concerns about the 10 warning signs proposed by the Jeffrey Modell Foundation have been raised, given signs pertaining to PIDD, such as sporadic infections, autoimmunity, autoinflammation and malignancy, are not accounted for.^(17-20)^ In this sample, signs associated with the immune dysregulation syndrome class of PIDD, other than those included in the Jeffrey Modell Foundation list were reported,^(21)^ such as cytopenia and gastrointestinal diseases. Some authors have proposed the inclusion of “autoimmunity” in the list of 10 warning signs developed by the Jeffrey Modell Foundation,^(22)^ based on findings of a retrospective study, in which this sign was associated with increased sensitivity regarding suspicion of PIDD in pediatric patients, although not in adults.^(18)^

As in a prior Brazilian study,^(10)^ most physicians in this sample reported having managed patients with a history of recurrent infections and frequent use of antibiotics. Also, more than half reported having suspected PIDD, a higher percentage relative to a previous national report (42%).^(23)^ In this study, 44.6% of physicians reported having ordered ancillary tests when dealing with PIDD patients, the most common being laboratory tests, such as serum immunoglobulin levels differential lymphocyte count. This finding is somewhat different from those of a study conducted with pediatricians in the United Arab Emirates. In that study, complete blood count and serum immunoglobulin levels were the most commonly reported tests (96%), followed by IgG subclass levels, and chest X-ray (76.5%).^(15)^ The recent introduction of immunology as a specialty training program may explain discrepant findings.

In this study, lack of access to laboratory tests was the most critical barrier to PIDD diagnosis. This finding is congruent with previous Latin American data.^(8,10)^ Other less common challenges reported were high test costs and shortage of specialists, which were rated higher in other studies.^(10)^ Overall, the number of specialists and reference centers has increased worldwide. However, specialized laboratories have not followed this trend.^(8)^

Antibody deficiency has been singled out as the most common PIDD in several studies^(6,12,23-25)^ and the diagnosis of this condition does not require complex laboratory infrastructure.^(26)^ Nevertheless, a Peruvian report including 13 national hospitals reported limited access to immunological tests, such as immunoglobulin levels (56.5%).^(23)^ Moreover, only four centers in Peru perform simple lymphocyte population analysis for PIDD. Although the INSN is the primary national pediatric reference center in Peru, research and private collaboration initiatives are the only settings where flow cytometry analyses can be conducted.^(27)^ Therefore, advanced flow cytometry studies, genomic analysis and other tests can only be carried out abroad.

### Limitations

This was the first study to investigate the level of PIDD awareness in Peru, and was conducted at a national pediatric reference center, namely the most important pediatric hospital providing specialized training to Peruvian pediatricians. Although more than half of physicians (residents and pediatricians) working at INSN were surveyed, convenience sampling was used instead of random sampling. Given physicians with particular interest in the topic were more likely to participate, results may have been biased towards higher levels of awareness. Therefore, it would not be surprising if actual levels of awareness were lower. Also, the type and number of courses or conferences physicians attended was not deeply investigated. Hence, the risk of memory bias must be accounted for.

### Clinical applicability

This study revealed a need for PIDD-specific education in residency programs in Peru. Training programs may contribute to increased awareness of PIDD, suspicion and referral of patients.^(26,28)^ According to a Mexican study, 75 trained physicians are required to get one patient referred.^(29)^ Structured training programs should aim to provide general knowledge about PIDD, as treatable diseases requiring early suspicion, and to inform physicians about referral of affected patients. Regular awareness campaigns should also be organized.

In Latin America, PIDD have some specific features. Hence, locally adapted warning signs have been proposed in Brazil.^(30)^ Physicians in Peru should be aware of endemic infectious diseases such as bartonellosis and dengue fever. The inclusion of bacillus Calmette-Guerin and oral poliomyelitis vaccines in the national immunization schedule introduces some clinical and laboratory features which are not shared by other cohorts.^(8)^ Hence, the development of PIDD warning signs adapted to the Peruvian reality is suggested.

## CONCLUSION

This study revealed limited awareness of primary immunodeficiency, diseases among physicians working at *Instituto Nacional de Salud del Niño*. Although most physicians suspected primary immunodeficiency diseases in patients with a history of recurrent infections and frequent use of antibiotics, not all of them were familiar with the 10 warning signs proposer by the Jeffrey Modell Foundation, nor were they able to describe the ancillary tests ordered in suspected cases. Enhanced training on primary immunodeficiency diseases, and improved structuring of residency programs is suggested to increase awareness of these disorders.

## Appendix 1

**Table:** Questionnaire about awareness of primary immunodeficiency diseases in pediatric residents and pediatricians

Introduction:	
The following survey is aimed to examine the level of awareness of primary immunodeficiency diseases among physicians working at *Instituto Nacional de Salud del Niño* (INSN) - Breña, LIM, Peru. This survey comprises two sections: sociodemographic data and data on awareness of primary immunodeficiency diseases. This survey is individualized, and will take a maximum of 10 minutes to complete.	
Instructions:	
Please read the questions carefully and select the answer you think is correct.	
Section 1: Sociodemographic data	
1. How old are you? _________	
2. What is your gender?	
______ Female	
______ Male	
3. What is the highest academic level you have achieved?	
______ Resident	
______ Specialist	
4. In which area are you doing or have you done your residency?	
______ Clinical	
______ Surgical	
5. At each of the following academic levels, how many years have you worked in that area?	
General practitioner: _____ years	
Resident: _____ years	
Specialist: _____ years	
6. a) Have you completed any rotations in immunology units (either during your residency, or as complementary training thereafter)?	
______ No (go to question 7)	
______ Yes	
6. b) In which unit did this rotation take place?	
_____ *Unidad Funcional de Alergia, Asma e Inmunologia* (UFAAI)	
_____ Other (specify): ______________________	
7. a ) What type of training did you receive as an undergraduate student? More than one alternative may be selected	
______ None	
______ Course on primary immunodeficiency diseases	
______ Rotation in an immunology unit in which patients with primary immunodeficiency diseases are managed	
______ Presentation addressing primary immunodeficiency diseases (conference or clinical case discussion)	
7. b) What type of training did you receive during your residency? More than one alternative may be selected	
_____ None	
______ Course on primary immunodeficiency diseases	
______ Rotation in an immunology unit in which patients with primary immunodeficiency diseases are managed	
______ Presentation addressing primary immunodeficiency diseases (conference or clinical case discussion)	
7. c) What type of training did you receive after having completed your residency? You can select more than one option	
______ None	
______ Course on primary immunodeficiency diseases	
______ Rotation in an immunology unit in which patients with primary immunodeficiency diseases are managed	
______ Presentation addressing primary immunodeficiency diseases (conference or clinical case discussion)	
Section 2. Awareness of primary immunodeficiency diseases	
8. Did you know patients who frequently use antibiotics may have a primary immunodeficiency disease? Consider frequent as two or more months on antibiotics, with poor clinical response, or need of intravenous antibiotics to control infections.	
______ No, never	
______ Yes, in some cases	
______ Yes, in approximately half of cases	
______ Yes, always	
9. Some physicians believe that primary immunodeficiency diseases are not treatable. Do you think primary immunodeficiency diseases are amenable to treatment?	
______ No, never	
______ Yes, in some cases	
______ Yes, in approximately half of cases	
______ Yes, always	
10. Do you think patients with a primary immunodeficiency disease suffer from a severe disease?	
______ No, never	
______ Yes, in some cases	
______ Yes, in approximately half of cases	
______ Yes, always	
11. a) Are you familiar with the 10 warning signs of primary immunodeficiency diseases?	
______ No (go to question 12)	
______ Yes	
11. b) Which are the 10 warning signs of primary immunodeficiency diseases? List them below	
12. What is the greatest challenge faced when examining patients with primary immunodeficiency diseases? Please select only one alternative.	
______ Lack of access to laboratory tests	
______ Shortage of specialists	
______ High test costs	
______ Other: ________________________________________	
13. Do you see patients with a history of recurrent infections? Consider recurrent infections as ≥4 new ear infections within one year, ≥2 serious sinus infections within one year, ≥2 pneumonias within one year, recurrent deep abscesses in skin or organ (*e.g*. liver, lungs), or ≥2 deep-seated infections (*e.g*. sepsis).	
______ No never	
______ Yes, ≥1 patient per year	
______ Yes, ≥1 patient per month	
______ Yes, ≥1 patient per week	
14. Do you see patients who frequently take antibiotics? Consider frequent as two or more months on antibiotics with poor response or need of intravenous antibiotics to control infections	
______ No, never	
______ Yes, ≥1 patient per year	
______ Yes, ≥1 patient per month	
______ Yes, ≥1 patient per week	
15. a) Have you ever evaluated a patient for primary immunodeficiency diseases?	
______ No (go to question 16)	
______ Yes	
15. b) What laboratory or imaging tests did you request? List them below:	
___________________________________________________________________	
16. a) When evaluating patients for primary immunodeficiency diseases, do you know who to contact?	
______ No (go to question 17)	
______ Yes	
16. b) List your contacts:	
17. Do you think it is safe to immunize patients with primary immunodeficiency diseases using the following types of vaccines?	
______ No	______ Yes
Live attenuated microorganisms	
__________	
Inactivated	
__________	

Thank you for having taken the time to answer this questionnaire.
