# Supplementary material for: Awareness of primary immunodeficiency diseases at a national pediatric reference center in Peru
Source: Einstein (Sao Paulo). 2021 Dec 3;19:eAO6289. doi: 10.31744/einstein_journal/2021AO6289 (PMC8664282; doi:10.31744/einstein_journal/2021AO6289)
Supplement: Translation

# Translation: Conscientização sobre imunodeficiências primárias em um centro nacional de referência pediátrica no Peru

**Authors:** Liz Eliana Veramendi-Espinoza, Jessica Hanae Zafra-Tanaka, Crhistian Toribio-Dionicio, Mariella R. Huamán, Gabriela Pérez, Wilmer Córdova-Calderón

**Affiliations:** 1 Peru Departamento de Alergia, Unidade de Imunodeficiência Primária, Hospital Nacional Edgardo Rebagliati Martins. Lima, LIM, Peru.; 2 Peru Centro de Excelência para Doenças Crônicas, Universidad Peruana Cayetano Heredia, Lima, LIM, Peru.; 3 Peru Faculdade de Medicina, Universidad Nacional Mayor de San Marcos, Lima, LIM, Peru.; 4 Peru Sociedad Cientifica de San Fernando, Universidad Nacional Mayor de San Marcos, Lima, LIM, Peru.; 5 Peru Unidad Funcional de Alergia, Asma e Inmunologia, Instituto Nacional de Salud del Niño, Breña, LIM, Peru.

**Keywords:** Conscientização, Educação médica, Doenças da imunodeficiência primária/diagnóstico, Peru

## Abstract

**Objetivo:**

Investigar o nível de conscientização sobre imunodeficiências primárias entre os médicos do *Instituto Nacional de Salud del Niño*.

**Métodos:**

Estudo transversal com todos os residentes de pediatria e pediatras que faziam parte do corpo clínico do *Instituto Nacional de Salud del Niño* quando o estudo foi realizado (2017-2019). Médicos atuantes na unidade de imunologia e nos setores de cirurgia foram excluídos. Foram estudados três aspectos da conscientização sobre imunodeficiências primárias: educação, conhecimento geral e suspeita diagnóstica e ações adotadas frente à suspeita.

**Resultados:**

A amostra foi composta por 83 médicos com mediana de idade de 33 anos, em sua maioria mulheres (71,1%) e metade residentes em pediatria. Durante a graduação, 43,1% relataram participação em cursos sobre imunodeficiências primárias, e 39,2% participação em conferências relacionadas. Durante o programa de residência, 25,9% relataram participação em cursos sobre imunodeficiências primárias, e 60,3% participação em conferências relacionadas. Entre os pediatras, 50% relataram participação em cursos sobre imunodeficiências primárias e 53,1% participação em conferências relacionadas. Apenas 39,8% dos médicos declararam conhecer os 10 sinais de alerta propostos pela Fundação Jeffrey Modell. Mais da metade dos médicos considerou a falta de acesso a exames laboratoriais o principal desafio no diagnóstico das imunodeficiências primárias.

**Conclusão:**

Este estudo revelou conscientização limitada sobre imunodeficiências primárias entre os médicos do corpo clínico do *Instituto Nacional de Salud del Niño*. Embora a maioria dos médicos tenha suspeitado de imunodeficiências primárias em pacientes com histórico de infecções recorrentes e uso frequente de antibióticos, nem todos conheciam os 10 sinais de alerta propostos pela Fundação Jeffrey Modell e nem todos foram capazes de descrever os exames complementares solicitados nos casos suspeitos.

## INTRODUÇÃO

As imunodeficiências primárias (IDP) são um grupo heterogêneo de doenças do sistema imune. As apresentações mais comuns incluem infecções recorrentes graves, neoplasias, atopias e condições autoimunes, com alta mortalidade, sobretudo em pacientes com imunodeficiência combinada grave.^(1,2)^ Aproximadamente 6 milhões de pessoas sofrem de IDP no mundo.^(3)^ Entretanto, o número de casos documentados é menor.

Em agosto de 2020, a Sociedade Latino Americana de Imunodeficiências (LASID) registrou 8.383 casos de IDP. O Peru contribuiu com 213 casos (2,5% do total), dos quais 33,8% foram relatados pela *Unidad Funcional de Alergia, Asma e Inmunologia* (UFAAI), do *Instituto Nacional de Salud del Niño* (INSN) - Breña, LIM, Peru^(4)^ o centro de saúde mais importante para especialização em pediatria no país. Em 2015, a Fundação Jeffrey Modell incluiu UFAAI e dois outros centros de imunologia entre os seus Centros de Diagnóstico e Pesquisa credenciados, um passo fundamental para o estabelecimento de colaborações internacionais para o benefício dos pacientes com IDP. Apesar do número crescente de casos registrados na América Latina anualmente, um reflexo dos avanços das técnicas diagnósticas e da maior conscientização,^(5,6)^ restam muitos desafios a vencer para garantir a cobertura de um número maior de pacientes com IDP.^(7,8)^

Diversos estudos sugerem que a falta de conscientização a respeito das IDP na classe médica pode atrasar o diagnóstico e tratamento dos pacientes afetados. Em um estudo realizado nos Estados Unidos, a conscientização sobre as IDP se limitou a 32% dos médicos e foi maior entre os pediatras.^(9)^ Em um estudo brasileiro, embora 80% dos médicos tenha admitido que infecções recorrentes podem ter relação com IDP, apenas 40% deles examinou esses pacientes.^(10)^ Analogamente, uma enquete investigando a conscientização sobre as IDP no Irã teve aprovação de apenas 32% dos médicos. Embora os escores mais altos tenham sido atribuídos ao tratamento, 86% dos médicos relatou dificuldades no manejo dos pacientes.^(11)^

Conhecimento e conscientização têm significados distintos, sendo essa mais relacionada à atitude e à ação.^(6,9-11)^ Nesse sentido, a falta de conscientização entre os médicos pode explicar o número limitado de casos de IDP relatados, e o atraso no diagnóstico em países da América Latina, como o Peru.^(4,12)^Este estudo teve por objetivo investigar o nível de conscientização sobre IDP entre médicos de um centro nacional de referência para doenças pediátricas no Peru. Os achados deste estudo podem servir de base para o desenvolvimento de estratégias voltadas para a conscientização sobre IDP, e contribuir para o diagnóstico precoce e a instituição oportuna de tratamento dos pacientes afetados.

## OBJETIVO

Investigar o nível de conscientização sobre imunodeficiências primárias entre os médicos do *Instituto Nacional de Salud del Niño*.

## MÉTODOS

### Desenho e população do estudo

Trata-se de um estudo transversal realizado no INSN. A população do estudo incluiu todos os médicos que faziam parte do quadro de profissionais do INSN durante o período experimental (outubro de 2017 a março de 2019). Os residentes em pediatria e pediatras que atuavam nas unidades de internação pediátrica foram incluídos. Os médicos que atuavam na UFAAI foram excluídos dado o seu treinamento formal em IDP. Os cirurgiões também foram excluídos devido ao acesso limitado a esses especialistas.

### Procedimentos

Inicialmente, foi empregada a amostragem aleatória estratificada por nível de especialização (residente de pediatria ou pediatra). A população do estudo incluiu 148 médicos (105 residentes e 43 pediatras). Empregando-se o software Epidat V. 4.0, foi estimado um tamanho amostral de 106 (75 residentes e 31 pediatras) para se obter uma proporção de 40,3% de médicos que já tinham examinado um paciente com IDP,^(10)^ com 5% de acurácia e um nível de significância de 5%.

Uma lista incluindo todos os médicos que faziam parte do quadro de profissionais do INSN no início do estudo foi utilizada para a identificação de possíveis participantes. Os médicos selecionados foram visitados duas vezes e convidados a participar. Porém diversos participantes em potencial se recusaram a responder ao questionário alegando falta de conhecimento do assunto, enquanto outros mostraram-se difíceis de contatar ou localizar. Por esses motivos, a amostragem por conveniência foi utilizada, e os médicos foram selecionados ao longo de cinco visitas ao complexo hospitalar.

### Variáveis do estudo

Três aspectos relacionados à conscientização sobre IDP foram investigados: educação, conhecimento geral, e suspeita diagnóstica e ações adotadas frente à suspeita.

Os aspectos educacionais incluíram perguntas sobre cursos, conferências, e estágios clínicos relacionados às IDP, realizados durante a gradução, residência, e ao longo da vida profissional.

O conhecimento geral foi avaliado por meio das seguintes perguntas: “Sabia que pacientes que tomam antibíoticos com frequência podem ter imunodeficiência primária?”, “Acha que as imunodeficiência primária podem ser tratadas?”, e “Acha que pacientes com imunodeficiência primária sofrem de uma doença grave?”. As alternativas de resposta foram “Não, nunca,” “Sim, em alguns casos,” “Sim, em aproximadamente metade dos casos,” e “Sim, sempre.”

Para avaliação da capacidade de suspeitar de IDP, os médicos foram convidados a listar os 10 sinais de alerta para IDP propostos pela Fundação Jeffrey Modell,^(13)^ avaliar cada um deles, e agrupá-los de acordo. As dificuldades encontradas pelos médicos no diagnóstico das IDP também foram exploradas. Os 10 sinais de alerta foram empregados por sua originalidade e escopo.^(6)^ Para melhor compreensão das ações adotadas frente à suspeita de IDP, os médicos foram questionados quanto à experiência em examinar pacientes com IDP, aos exames de laboratório e de imagem solicitados e aos contatos para consultoria.

Foram mensuradas também as seguintes variáveis sociodemográficas: idade, sexo, grau de especialização (residente em pediatria ou pediatra) e anos de estudo.

### Instrumento

Um questionário *ad hoc* contendo perguntas relacionadas às variáveis sociodemográficas e às IDP (educação, conhecimento geral e suspeita diagnóstica, e ações frente à suspeita) foi desenvolvido. O questionário foi validado por quatro especialistas em IDP e um estatístico, e testado em entrevistas com médicos peruanos (residentes em pediatria e pediatras) que não participaram do estudo, cujas sugestões foram consideradas na elaboração da versão final (Apêndice 1).

### Plano de análise de dados

As variáveis categóricas foram descritas como frequências absolutas e relativas. As variáveis quantitativas foram descritas por meio de medidas de tendência central e dispersão. As análises estatísticas foram realizadas empregando-se o software Stata v14 (StataCorp LP, 2015, College Station, Texas, EUA), com um nível de significância de 5%.

### Aspectos éticos

Este estudo foi revisado e aprovado pelo Comitê de Ética em Pesquisa do INSN numéro 0287-2018. Os participantes receberam um termo de consentimento livre e informado, contendo as explicações sobre os objetivos do estudo ao serem convidados a participar. As informações coletadas foram mantidas em sigilo.

## RESULTADOS

Esta amostra foi composta por 83 médicos com mediana de idade de 33 anos. A maioria dos médicos selecionados era do sexo feminino (71,1%), sendo a metade residentes em pediatria. No que se refere à educação recebida sobre IDP durante a graduação, 43,1% relataram participação em cursos específicos, 39,2% participação em conferências relacionadas, e apenas 2% estagiaram em unidade de imunologia. No que se refere ao programa de residência, 25,9% relataram participação em cursos específicos sobre IDP, 60,3% participação em conferências relacionadas e apenas 3,5% estagiaram em unidade de imunologia.

Os pediatras foram questionados sobre a educação continuada (ou seja, educação após a conclusão da residência em pediatria). Nesse grupo, 50% relataram participação em cursos específicos sobre IDP, 53,1% participação em conferências relacionadas, e apenas 3,1% estagiaram em unidade de imunologia. O conhecimento geral sobre IDP entre os médicos está descrito na tabela 1.

**Tabela 1:** Conhecimento geral sobre imunodeficiências primárias

	Nunca n (%)	Alguns casos n (%)	Aproximadamente metade n (%)	Sempre n (%)
Sabia que pacientes que tomam antibióticos com frequência podem ter imunodeficiência primária?	4 (4,8)	55 (66,3)	20 (24,1)	4 (4,8)
Acha que as imunodeficiência primária podem ser tratadas?	5 (6,1)	42 (51,2)	24 (29,3)	11 (13,4)
Acha que pacientes com imunodeficiência primária sofrem de uma doença grave?	1 (1,2)	35 (43,2)	28 (34,6)	17 (20,9)

IDP: imunodeficiência primária.

Nesta amostra, 39,8% (n=33) dos médicos relataram conhecer os 10 sinais de alerta propostos pela Fundação Jeffrey Modell^(13)^ (Tabela 2). Os sinais relacionados à infecção foram descritos com maior frequência, e 33,7% (n=28) dos médicos identificaram outros sinais, incluindo infecções recorrentes e oportunistas (78,6%, n=22), doenças gastrointestinais não especificadas (35,7%, n=10), e doenças hematológicas, como a citopenia autoimune (21,4%, n=6). Além disso, 94,9% (n= 74) dos médicos declararam que vacinas com patógenos vivos devem ser evitadas em pacientes com IDP, e 92,6% (n=75) que vacinas inativadas são seguras.

**Tabela 2:** Lista de 10 sinais de alerta (Fundação Jeffrey Modell) relatados pelos médicos

Sinal de alerta	n (%)
≥4 novas infecções de ouvido em um ano	23 (69,7)
≥2 pneumonias em um ano	20 (60,6)
Abscessos recorrentes profundos na pele ou em órgãos (por ex.: fígado, pulmões)	19 (57,6)
≥2 sinusites graves em um ano	17 (51,5)
Lactentes com dificuldade de ganho de peso ou crescimento	17 (51,5)
≥2 infecções profundas (por ex.: sepse, meningite)	16 (48,5)
≥2 meses em uso de antibióticos com pouca resposta	12 (36,4)
História familiar de imunodeficiência primária	9 (27,3)
Necessidade de antibioticoterapia intravenosa para debelar infecções	5 (15,2)
Candidíase oral ou infeção fúngica cutânea persistente	3 (9,1)

A maioria dos médicos relatou ter atendido pacientes com histórico de infecções recorrentes (96,4%, n=80) e uso frequente de antibióticos (92,8%, n=77) (Tabela 3). Mais da metade dos médicos nesta amostra (64,6%, n=53) relataram ter suspeitado de IDP e solicitados exames complementares. Entretanto, apenas 69,8% (n=37) foram capazes de descrever os exames solicitados (Tabela 4). Com relação às ações adotadas frente à suspeita de IDP, 74,7% (n=62) dos médicos relataram ter encaminhado os pacientes para um especialista ou instituição especializada. Em todos os casos, a UFAAI foi a unidade escolhida para encaminhamento, seguida por outros hospitais (9,7%, n=6) e outros especialistas (11,3%, n=7).

**Tabela 3:** Experiência prática geral relatada pelos médicos

	Nunca n (%)	≥1/ano n (%)	≥1/mês n (%)	≥1/semana n (%)
Atende pacientes com infecções recorrentes?	3 (3,6)	36 (43,4)	36 (43,4)	8 (9,6)
Atende pacientes que usam antibióticos com frequência?	6 (7,2)	32 (38,6)	35 (42,2)	10 (12,1)

**Tabela 4:** Exames complementares solicitados pelos médicos

Exame complementar	n (%)
Imunoglobulinas	36 (97,3)
Contagem de linfócitos	33 (89,2)
Hemograma completo	21 (56,8)
Radiografias, TC, RM	12 (32,4)
Cultura/biópsia	5 (13,5)
Testagem para HIV	4 (10,8)
Metabolismo oxidativo dos neutrófilos	4 (10,8)
Dosagem de complemento	3 (8,1)
Sequenciamento do exoma	2 (5,4)
Outros	19 (51,4)

TC: tomografia computadorizada; RM: ressonância magnética; HIV: vírus da imunodeficiência humana.

Ao serem questionados sobre os desafios enfrentados no diagnóstico de IDP, os médicos apontaram a falta de acesso a exames laboratoriais como o principal (57,7%), seguida pelo alto custo dos exames (32,1%), e pela falta de especialistas em imunologia (5,1%).

## DISCUSSÃO

Nesta amostra, menos da metade dos participantes relatou participação em cursos e conferências ou estágios em unidade de imunologia durante a graduação. O número de médicos com participação eventos relacionados à IDP aumentou durante e após a residência. De forma geral, o número de estágios realizados em unidades de imunologia foi baixo. Em contrapartida, um estudo prévio envolvendo 27 hospitais gerais no Brasil mostrou que os pediatras aprendiam sobre IDP durante ou após a residência (72,2% e 75,9%, respectivamente).^(10)^ A imunologia é uma especialidade médica nova no Peru e a UFAAI foi inaugurada recentemente, em 2012. Esses fatores podem explicar a diferença entre os estudos, uma vez que os médicos treinados antes de 2021 podem não ter tido acesso a uma educação adequada sobre IDP.

Este estudo revelou que o conhecimento dos médicos sobre IDP é limitado. Achados semelhantes foram relatados em estudos prévios que avaliaram o conhecimento específico sobre IDP em diferentes países, tais como os Estados Unidos, o Irã, os Emirados Árabes Unidos e o México. Esses estudos apontaram níveis baixos de informação entre clínicos gerais, médicos que atuam na atenção básica e pediatras, inclusive residentes.^(9,14-16)^ Curiosamente, esses estudos datam de 5 a 10 anos, o que indica que se trata de um problema persistente em diversos países, independente da condição socioeconômica.

Quanto aos sinais de alerta, os associados à infecção, que são característicos da IDP, foram identificados com maior frequência.^(1)^ Entretanto, os 10 sinais de alerta propostos pela Fundação Jeffrey Modell foram alvo de diversos questionamentos, pois não consideram o fato de que as IDP incluem enfermidades caracterizadas por infecções esporádicas, autoimunidade, autoinflamação e malignidade.^(17-20)^ Nesta amostra, sinais relacionados às IDPs caracterizadas como síndrome de desregulação imune, que diferem dos listados pela Fundação Jeffrey Modell, foram mencionados,^(21)^ tais como citopenia e doenças gastrointestinais. Alguns autores propuseram a inclusão de “autoimunidade” na lista dos 10 sinais de alerta da Fundação Jeffrey Modell,^(22)^ com base nos achados de um estudo retrospectivo, em que esse sinal se mostrou associado à maior sensibilidade na formulação de hipótese diagnóstica de IDP em pacientes pediátricos, embora não em adultos.^(18)^

Assim como em um estudo brasileiro anterior,^(10)^a maioria dos médicos incluídos nesta amostra relataram atender pacientes com história de infecções recorrentes e uso frequente de antibióticos. Mais da metade relataram ainda ter suspeitado de IDP, uma percentagem mais alta em relação a um relato nacional prévio (42%).^(23)^ Neste estudo, 44,6% dos médicos declararam ter solicitado exames complementares ao atender pacientes com IDP, principalmente exames laboratoriais como dosagem sérica de imunoglobulinas e contagem diferencial de linfócitos. Esse achado difere ligeiramente dos relatados em um estudo realizado com pediatras nos Emirados Árabes Unidos, no qual o hemograma completo e a dosagem sérica de imunoglobulinas foram os exames mais solicitados (96%), seguidos pela dosagem de subclasses de IgG e pela radiografia de tórax (76,5%).^(15)^ A introdução recente da imunologia entre os programas de especialização pode explicar essas discrepâncias.

Neste estudo, a falta de acesso a exames laboratoriais foi a apontada como a principal barreira ao diagnóstico de IDP. Esse achado corrobora outros dados latino-americanos prévios.^(8,10)^ Outros desafios, menos comuns, foram o alto custo dos exames e a falta de especialistas, fatores esses que receberam maior destaque em outros estudos prévios.^(10)^ De forma geral, o número de especialistas e de centros de referência aumentou no mundo todo. Entretanto, os laboratórios especializados não seguiram essa tendência.^(8)^

A deficiência de anticorpos foi destacada como a causa mais comum de IDP em diversos estudos^(6,12,23-25)^ e o diagnóstico dessa condição não requer infraestrutura laboratorial complexa.^(26)^ Ainda assim, um estudo peruano que incluiu 13 hospitais nacionais revelou acesso limitado a exames como a dosagem de imunoglobulinas (56,5%).^(23)^ Além disso, apenas quatro centros no Peru disponibilizam análise de populações de linfócitos para IDP. Embora o INSN seja o principal centro de referência em pediatria do Peru, as análises por citometria de fluxo se limitam às pesquisas e colaborações com a iniciativa privada.^(27)^ Portanto, estudo avançados com citometria de fluxo, análise genômica e outros exames só podem ser realizados fora do país.

### Limitações

Esse estudo foi o primeiro a investigar o nível de conscientização sobre IDP no Peru, e realizado em um centro nacional de referência em pediatria, que é o principal hospital pediátrico que oferece especialização nessa área médica. Embora mais da metade dos médicos (residentes e pediatras) do corpo clínico do INSN tenham sido entrevistados, empregou-se a amostragem por conveniência em vez da amostragem aleatória. O fato de que médicos com interesse específico no assunto tinham maior probabilidade de participar pode ter introduzido um viés nos resultados, a favor da maior conscientização. Portanto, é perfeitamente possível que os níveis reais de conscientização sejam inferiores. Além disso, o tipo e o número de cursos e conferências não foram investigados a fundo, devendo-se considerar o risco de viés de memória.

### Aplicabilidade clínica

Este estudo revelou a necessidade de educação a respeito de IDP nos programas de residência no Peru. Programas de especialização podem contribuir para maior conscientização, suspeita e encaminhamento.^(26,28)^ Segundo um estudo mexicano, são necessários 75 médicos treinados para que um paciente seja encaminhado.^(29)^ Programas estruturados de especialização devem se voltar para propiciar o conhecimento geral sobre IDP, como doenças que podem ser tratadas que requerem suspeita diagnóstica precoce, e informar os médicos quanto ao encaminhamento dos pacientes, além da organização de campanhas regulares de conscientização.

As IDP têm algumas características específicas na América Latina. Por esse motivo, sinais de alerta adaptados à realidade local foram propostos no Brasil.^(30)^ Os médicos peruanos devem estar a par de doenças infecciosas endêmicas como a bartonelose e a dengue. A inclusão da vacina bacilo de Calmette-Guérin e da vacina oral contra poliomielite no calendário nacional de imunização agrega algumas características clínicas e laboratoriais que não se aplicam a outras coortes.^(8)^ Portanto, sugere-se a elaboração de uma lista de sinais de alerta adaptada à realidade peruana.

## CONCLUSÃO

Este estudo revelou conscientização limitada sobre imunodeficiências primárias entre os médicos do corpo clínico do *Instituto Nacional de Salud del Niño*. Embora a maioria dos médicos suspeite de imunodeficiências primárias em pacientes com histórico de infecções recorrentes e uso frequente de antibióticos, nem todos se estavam a par da lista de 10 sinais de alerta proposta pela Fundação Jeffrey Modell, e muitos não foram capazes de descrever os exames complementares solicitados em casos suspeitos. Sugere-se o aprimoramento dos programas de especialização em imunodeficiências primárias, e a estruturação dos programas de residência a fim de aumentar a conscientização a respeito dessas doenças.

## Apêndice 1

**Table:** Questionário de conscientização sobre imunodeficiências primárias entre residentes de pediatria e pediatras

Introdução:	
A enquete abaixo tem por objetivo avaliar o nível de conscientização sobre imunodeficiências primárias entre os médicos do corpo clínico do *Instituto Nacional de Salud del Niño* (INSN) - Breña, LIM, Peru. A enquete se divide em duas seções: dados sociodemográficos e dados referentes à conscientização sobre imunodeficiências primárias. Este enquete é pessoal, e leva, no máximo, 10 minutos para ser completada.	
Instruções:	
Por favor, leia as perguntas com atenção e selecione a resposta que lhe parecer correta.	
Seção 1: Dados sociodemográficos	
1. Qual a sua idade? ________________________	
2. Qual o seu gênero?	
______ Feminino	
______ Masculino	
3. Qual o grau acadêmico mais alto que atingiu?	
______ Residente	
______ Especialista	
4. Em que área está fazendo ou fez a sua residência?	
______ Área clínica	
______ Área cirúrgica	
5. Considerando os graus acadêmicos abaixo, quanto anos trabalhou na área escolhida?	
Clínico geral: _____ anos	
Residente: _____ anos	
Especialista: _____ anos	
6. a) Estagiou em unidades de imunologia (durante sua residência ou como treinamento complementar depois)?	
______ Não (vá para a pergunta 7)	
______ Sim	
6. b) Em qual unidade realizou o estágio?	
_____ *Unidad Funcional de Alergia, Asma e Inmunologia* (UFAAI)	
_____ Outra (especificar): ______________________	
7. a) Que tipo de treinamento recebeu durante a graduação? Mais de uma alternativa pode ser selecionada	
______ Nenhum	
______ Curso sobre imunodeficiências primárias	
______ Estágio em unidade de imunologia que recebe pacientes com imunodeficiências primárias para tratamento	
______ Apresentação que abordou imunodeficiências primárias (conferência ou discussão de caso clínico)	
7. b) Que tipo de treinamento recebeu durante a residência? Mais de uma alternativa pode ser selecionada	
_____ Nenhum	
______ Curso sobre imunodeficiências primárias	
______ Estágio em unidade de imunologia que recebe pacientes com imunodeficiências primárias para tratamento	
______ Apresentação que abordou imunodeficiências primárias (conferência ou discussão de caso clínico)	
7. c) Que tipo de treinamento recebeu após a residência? Mais de uma alternativa pode ser selecionada	
______ Nenhum	
______ Curso sobre imunodeficiências primárias	
______ Estágio em unidade de imunologia que recebe pacientes com imunodeficiências primárias para tratamento	
______ Apresentação que abordou imunodeficiências primárias (conferência ou discussão de caso clínico)	
Seção 2. Conscientização sobre imunodeficiências primárias	
8. Sabia que pacientes que usam antibióticos com frequência podem ter imunodeficiência primária? Considere frequente como dois ou mais meses de tratamento com antibióticos, com resposta clínica inadequada, ou necessidade de antibioticoterapia intravenosa para controle de infecções.	
______ Não, nunca	
______ Sim, em alguns casos	
______ Sim, em aproximadamente metade dos casos	
______ Sim, sempre	
9. Alguns médicos acreditam que as imunodeficiências primárias não são tratáveis. Acha que as imunodeficiências primárias podem ser tratadas?	
______ Não, nunca	
______ Sim, em alguns casos	
______ Sim, em aproximadamente metade dos casos	
______ Sim, sempre	
10. Acha que pacientes com imunodeficiência primária têm uma doença grave?	
______ Não, nunca	
______ Sim, em alguns casos	
______ Sim, em aproximadamente metade dos casos	
______ Sim, sempre	
11.a) Conhece os 10 sinais de alerta para imunodeficiências primárias?	
______ Não (vá para a pergunta 12)	
______ sim	
11. b) Quais são os 10 sinais de alerta para imunodeficiências primárias? Liste os sinais abaixo	
12. Qual o maior desafio encontrado no exame de pacientes com imunodeficiências primárias? Por favor, selecione apenas uma alternativa.	
______ Falta de acesso a exames laboratoriais	
______ Falta de especialistas	
______ Alto custo dos exames	
______ Outros: ________________________________________	
13. Atende pacientes com infecções recorrentes? Considere infecções recorrentes como ≥4 novas infecções de ouvido em um ano, ≥ 2 sinusites graves em um ano, ≥ 2 pneumonias em um ano, abscessos profundos recorrentes na pele ou em órgãos (por ex., fígado, pulmões) ou ≥2 infecções profundas (por ex., sepse)	
______ Não, nunca	
______ Sim, ≥1 paciente por ano	
______ Sim, ≥1 paciente por mês	
______ Sim, ≥1 paciente por semana	
14. Atende pacientes com história de uso frequente de antibióticos? Considere frequente como dois ou mais meses de tratamento com antibióticos, com resposta inadequada, ou necessidade de antibioticoterapia intravenosa para controle de infecções.	
______ Não, nunca	
______ Sim, ≥1 paciente por ano	
______ Sim, ≥1 paciente por mês	
______ Sim, ≥1 paciente por semana	
15. a) Já examinou pacientes com suspeita de imunodeficiência primária?	
______ Não (vá para a pergunta 16)	
______ Sim	
15. b) Quais exames laboratoriais ou de imagem solicitou? Liste os exames abaixo:	
___________________________________________________________________	
16. a) Ao examinar pacientes com suspeita de imunodeficiência primária, sabe quem contatar?	
______ Não (vá para a pergunta 17)	
______ Sim	
16. b) Forneça os detalhes de seus contatos:	
17. Considera segura a imunização de pacientes com imunodeficiências primárias usando os tipos de vacinas abaixo?	
______ Não	______ Sim
Vacinas com micro-organismos vivos atenuados	
__________	
Inativadas	
__________	

Obrigado pelo tempo dedicado a responder a esse questionário.
